# The Protective Effect of Heat-Inactivated *Companilactobacillus crustorum* on Dextran Sulfate Sodium-Induced Ulcerative Colitis in Mice

**DOI:** 10.3390/nu15122746

**Published:** 2023-06-14

**Authors:** Yujie Zhong, Tao Wang, Xin Wang, Xin Lü

**Affiliations:** 1 College of Food Science and Engineering, Northwest Agriculture and Forestry University, Yangling, Xianyang 712100, China; zyj0048@163.com (Y.Z.); wtao_2017@163.com (T.W.); wangxin_2018@nwsuaf.edu.cn (X.W.); 2 Faculty of Food Science and Engineering, Kunming University of Science and Technology, Kunming 650500, China

**Keywords:** postbiotics, inflammation, gut barrier damage, oxidative damage, gut microbiota

## Abstract

Heat-inactivated microorganisms are a typical class of postbiotics with promising potential health effects, as they contain various physiologically active components. Dietary supplementation with *Companilactobacillus crustorum* MN047 (CC) has been shown to have the potential to alleviate ulcerative colitis (UC). However, it is unclear whether the UC-relieving effect of this strain is partly attributed to its bacterial composition. Therefore, the interventional effects of heat-inactivated CC (HICC) on UC mice were explored. The results showed that the administration of HICC significantly ameliorated the UC-related pathological parameters by (1) alleviating the pathologic lesions of UC (e.g., preventing the increase in disease activity index and the shortening of colon length); (2) ameliorating the colonic inflammation (e.g., inhibiting the expressions of chemokines and pro-inflammatory cytokines, such as *Cxcl1*, *Cxcl5*, *Ccl7*, *TNF-α*, *IL-1β*, *IL-6*, and *MCP-1*; (3) attenuating the oxidative damage (e.g., suppressing the increase in myeloperoxidase and malondialdehyde); (4) mitigating the damage of gut barrier (e.g., promoting colonic occludin, ZO-1, and claudin levels); and (5) modulating gut microbiota structure (e.g., increasing the relative abundance of potential probiotics, such as *Akkermansia* and *Lactobacillus*). In conclusion, our study suggested that HICC can be effective in preventing UC and has the potential as a dietary supplement to intervene in UC.

## 1. Introduction

Ulcerative colitis (UC) is an immune-mediated chronic inflammatory bowel disease (IBD). It has become a globally disease with increasing prevalence, especially in emerging industrialized countries such as South America, Asia, Africa, and the Middle East [1]. Long-term UC may increase the risk of colorectal cancer [2]. A meta-analysis has shown that the cumulative risk of colorectal cancer during the first, second, and third decade of UC is 1.5%, 7.2%, and 23.6%, respectively, which is consistent with the risk observed in both Asia and West populations [3]. Therefore, UC severely affects the quality of human life. Currently, the main interventions for UC are pharmacological treatments, which mainly include aminosalicylic acid [4], hydrocortisone [5], and metronidazole [6]. As these drugs can cause a variety of side effects, some natural safety emerging interventions have attracted wide attention.

The pathogenesis of UC is complex and involves various factors, including the dysbiosis of intestinal microbiota, impairment of the intestinal barrier, and chronic oxidative damage [7]. Intestinal microbiota dysbiosis is a key contributing element to UC. It will result in a lesion of the epithelial barrier of intestine, which can directly affect intestinal luminal permeability and eventually result in the development of UC [8]. The gut barrier integrity is a prerequisite for maintaining a stable intestinal environment. Studies have shown that defects in the intestinal mucosa barrier can cause an increase in intestinal permeability, allowing for unrestricted passage of intestinal contents and microorganisms through the intestinal lamina propria, ultimately triggering intestinal inflammation and UC [9,10]. Probiotic supplementation is an efficacious way to modulate the balance of intestinal microbiota and maintain the integrity of the intestinal barrier [11,12]. In recent years, some probiotics have already been involved in the mitigation of UC such as *Lactobacillus rhamnosus* SHA113 [13], *Pediococcus pentosaceus* CECT 8330 [14], and *Bacillus cereus* HMPM18123 [15]. According to the definition, probiotics are required to be alive and maintain a certain number of live bacteria when they reach the gastrointestinal tract of the host [16]. However, most probiotic preparations contain a significant number of dead or injured microorganisms, especially in the case of end-of-life products [17]. These non-viable microorganisms and their components also possess noteworthy physiological activities that have often been overlooked. Moreover, these non-viable microorganisms, also known as postbiotics, have significant benefits compared with probiotics (e.g., stability, safety, and better processability) [18]. Thus, they are suitable for infants with weakened immunity and people with impaired intestinal barriers.

Nowadays, scientific evidence has suggested that various inactivated microorganisms can alleviate UC. For example, heat-inactivated *L. acidophilus* PIN7 can alleviate dextran sulfate sodium (DSS)-induced UC by modulating immune-modulatory Toll-like receptor (TLR) 6 signaling and maintaining gut microbiota balance [19]. Heat-killed *Lactobacillus plantarum* Zhang-LL can relieve the symptoms of UC in rats by reducing the serum levels of pro-inflammatory cytokines (e.g., *TNF-α* and *INF-γ*), decreasing gut permeability by increasing the levels of tight-junctions (TJs) proteins (e.g., ZO-1, claudin, and occludin), and modulating gut microbiota [20]. In addition, heat-killed *Lactobacillus rhamnosus HN001* could improve DSS-induced UC by inhibiting oxidative stress [21]. Taken together, it can be inferred that the inactivated microorganisms exert protective effects on UC through the regulation of intestinal microbiota, reinforcement of the gut barrier function, attenuation of the inflammatory response, and mitigation of oxidative damage.

*Companilactobacillus crustorum* MN047 (CC) is a potential probiotic previously isolated from the traditional fermented koumiss from Xinjiang (China). A previous study has suggested that the administration of CC can alleviate DSS-induced UC by maintaining the structure of gut microbiota [22]. However, it is unclear whether the UC mitigating effect of CC is partly attributed to its inactivated cells containing physiologically active substances. To solve this problem, this study is going to explore the interventional effect of heat-inactivated CC (HICC) on DSS-induced UC in mice. More specifically, the effect of HICC on the conventional pathological indicators, gut microbiota, intestinal barrier function, and oxidative damage was investigated. This study will further elucidate the mechanism of CC in alleviating UC.

## 2. Materials and Methods

### 2.1. The Preparation of HICC

The CC (no. 13163 in CGMCC) was cultured in de Man, Rogosa, and Sharpe (MRS) medium for 16 h at 37 °C. The CC bacterial cells were collected by centrifugation (6500× *g*, 5 min). After washing twice with 0.9% NaCl physiological saline solution (PSS), the CC was resuspended in PSS with a concentration of ~5 × 10^9^ CFU/mL and then heat-inactivated at 95 °C for 30 min to prepare HICC. The HICC was stored at −20 °C for subsequent dietary intervention in DSS-induced UC mice.

### 2.2. Animals and Treatment

The forty male C57BL/6 mice with an initial body weight of 20 ± 2 g were provided by the Hunan SJA Laboratory Animal (Changsha, Hunan, China). The experimental mice were housed in a controlled environment with 23 ± 2 °C and 12 h light/dark cycles. After 1-week adaptation, the mice were randomly assigned to four groups (n = 10, each group). Briefly, (1) healthy control group (Ctrl), (2) DSS-induced UC group (DSS), (3) HICC-treated UC group (HICC), and (4) sulfasalazine-treated UC group (SASP). Except for the Ctrl group, mice in the other three groups were provided sequentially with normal drinking water from days 0 to 3, 2.5% DSS (MW 36–50 kDa, MP Biomedicals, Aurora, OH, USA) dissolved in drinking water from days 4 to 10, and normal drinking water from days 11 to 14. Throughout the entire experimental process, mice in the HICC group and SASP group were administrated with HICC (200 μL/mouse/day, ~10^9^ CFU) and SASP (400 mg/kg body weight) by gavage, respectively, whereas mice in the Ctrl and DSS groups were gavaged with PSS (200 μL/mouse/day). Finally, the mice were euthanized by intraperitoneal administration of 100 mg/kg ketamine and 10 mg/kg xylazine (Sigma-Aldrich, St. Louis, MI, USA). The immune organs (thymus and spleen) were collected for weighing to assess immune organ indexes. The colonic tissue and mouse serum were stored at −80 °C for further biochemical or pathological analysis. All animal protocols were approved by the Animal Ethics Committee of Xi’an Jiaotong University (Permission no. SCXK 2018-001).

### 2.3. The Measurement of Disease Activity Index (DAI)

The standards for DAI scoring referred to a previous study [23]. The DAI score was calculated by combining three aspects: body weight, stool consistency, and fecal occult blood. The commercial fecal occult blood test kit was obtained from the Nanjing Jiancheng Bioengineering Institute (Nanjing, China).

### 2.4. Histological Analysis

The distal colonic tissues were collected and fixed with 4% paraformaldehyde for 24 h at 4 °C. The colonic samples were processed, including the preparation of paraffin sections, sectioning (5 μm), dewaxing, and rehydration of sections. Subsequently, the sections were stained with hematoxylin and esion (H and E) and Alcian blue staining. Finally, an Olympus microscope (Olympus Corporation, Shinjuku, Tokyo, Japan) was used to observe and take images. 

### 2.5. Immunofluorescence Assay

The immunofluorescence assay was used to measure the levels of ZO-1, occludin, and claudin-1 in colonic tissues. The detailed methods were described previously [22]. The primary antibodies included ZO-1 (product code: PB9234, Boster, Wuhan, China), claudin-1 (product code: WL03073, Wanleibio, Shenyang, China), and Occludin (product code: WL01996, Wanleibio). The FITC-conjugated secondary antibody (green, product code: WLA032) was purchased from Wanleibio.

### 2.6. RT-qPCR Assay

The extraction of total RNA, the synthesis of cDNA, and the amplification of quantitative PCR were performed as described previously [24]. The gene changes were calculated by the 2^−ΔΔCt^ method and normalized by the *GAPDH* gene. The specific primers are shown in Table S1.

### 2.7. The Analysis of Gut Microbiota

The gut microbiota of the mice were measured and analyzed as previously described [25]. Briefly, the PowerSoil DNA isolation kit (Mo Bio Laboratories, Carlsbad, CA, USA) was used to collect bacterial DNA in mouse feces. After executing the quality and quantity analyses, the amplification, purification, and sequencing of bacterial 16S rRNA (V3-V4 region) were performed by the Biomarker Technologies Corp (Beijing, China). The BMK Cloud Platform (https://international.biocloud.net, accessed on 21 January 2022) was used to perform data analysis. The specific bioinformatics analysis methods were fully consistent with our previous study [25].

### 2.8. The Determination of Biochemical Parameters

The levels of lipopolysaccharides (LPS) in mouse serum and the colonic expressions of TNF-α, IL-1β, and IL-6 in mouse colonic tissues were measured by the commercial ELISA kits purchased from Jingmei Biotech (Yancheng, Jiangsu, China). The levels of myeloperoxidase (MPO), malondialdehyde (MDA), superoxide dismutase (SOD), reduced glutathione (GSH), and total mercapto (T-SH) in mouse serum and/or colon were detected according to the requirements of commercial reagent kits (Nanjing Jiancheng Bioengineering Institute).

### 2.9. Statistical Analyses

Data were expressed as mean ± standard deviation (SD). GraphPad Prism 8 software was used for drawing and significance analysis. Significance analysis was performed using a one-way analysis of variance (ANOVA), followed by Tukey’s test for multiple comparisons. *p* value less than 0.05 was considered statistically significant. *, **, and *** indicated *p* < 0.05, 0.01, and 0.001, respectively. The relationship between gut microbiota and UC-related pathological parameters based on Pearson correlation coefficients analysis was performed using RStudio software (version 2022.12.0+353).

## 3. Results

### 3.1. HICC Alleviated the Pathological Symptoms in DSS-Induced UC Mice

**Figure 1:**
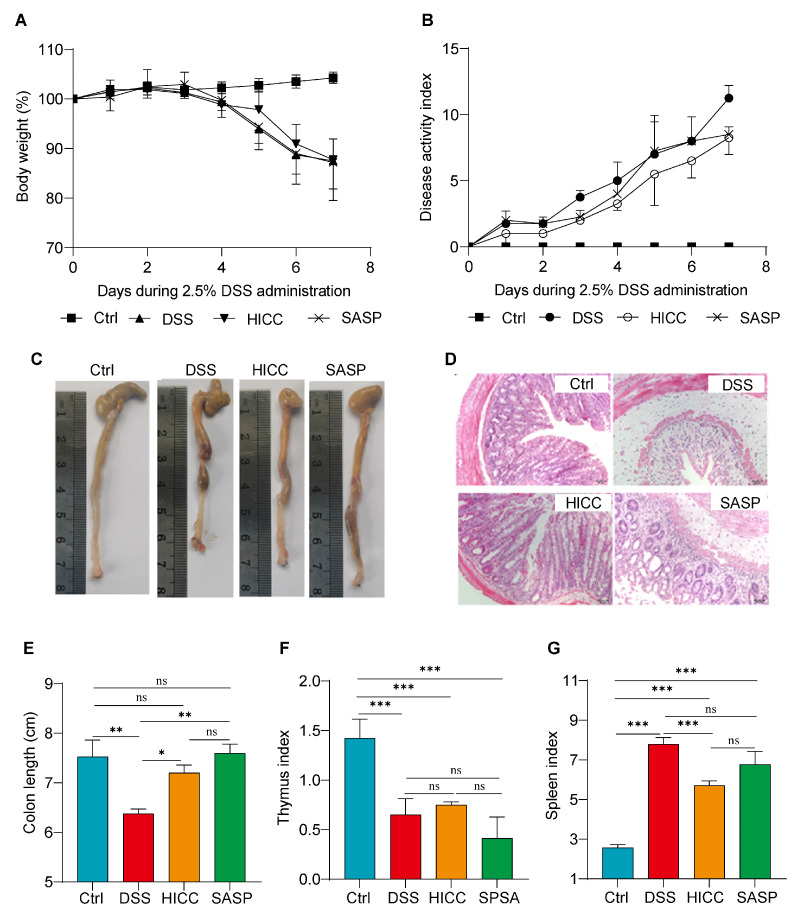
HICC alleviated the pathological symptoms in mice. (**A**) The body weight of mice during the period of DSS induction. (**B**) The DAI of mice during the period of DSS induction. (**C**) The representative colon images at the end of experiment. (**D**) The representative images of H and E staining of the colon in mice. (**E**) The colon length of mice. (**F**) The thymus index of mice. (**G**) The spleen index of mice. Data were expressed as mean ± SD (n = 10). *, **, and *** show *p* < 0.05, *p* < 0.01, and *p* < 0.001, respectively. ns represents no significant difference.

As UC is a typical IBD, suppressing the inflammatory response is an effective way to alleviate UC. To preliminary assess the anti-inflammatory action of HICC, the expressions of inflammation-related genes (*TNF-α*, *IL-6*, and *IL-1β*) were measured in inflammatory RAW 264.7 cells induced by LPS. It was found that the mRNA levels of *IL-6* and *IL-1β* were greatly downregulated after the treatment with cell fragments of CC (Figure S1), suggesting that HICC has the potential to mitigate UC. Therefore, the present study further investigated whether HICC had an intervention effect on DSS-induced UC in mice. During the DSS induction period, the mice in the DSS and SASP groups showed a significant decrease in body weight compared to the Ctrl group. However, the body weight loss was attenuated in the HICC intervention group on days five and six of DSS induction (Figure 1A). DSS treatment induced an increase in the DAI from days one to seven, whereas HICC intervention could partially alleviate it (Figure 1B). In addition, dietary intervention with HICC significantly alleviated the shortening of the colonic length and the increase in spleen index in the DSS-induced group (Figure 1C,E,G). However, there was no significant alleviative effect of HICC on the decrease in thymus index induced by DSS (Figure 1F). The results of H and E staining showed that DSS induction resulted in significant inflammatory cell infiltration, intestinal mucosal damage, and crypt defects in mouse colonic tissue. Dietary supplementation with HICC or SASP improved all these pathological changes, and the remission effect of HICC was better than SASP treatment (Figure 1D). Therefore, HICC could relieve intestinal lesions in UC mice.

### 3.2. HICC Ameliorated Intestinal Inflammation in Mice

**Figure 2:**
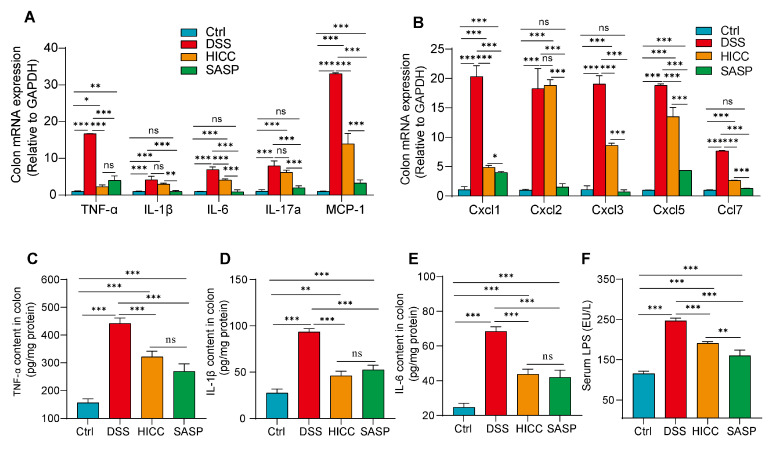
HICC alleviated intestinal inflammation in UC mice. (**A**) The gene expressions of inflammatory factors in the colon of DSS-induced UC mice. (**B**) The gene expressions of chemokines. (**C**) TNF-α content in the colon of DSS-induced UC mice. (**D**) IL-1β content in the colon of DSS-induced UC mice. (**E**) IL-6 content in the colon of DSS-induced UC mice. (**F**) Serum LPS content. Data were expressed as mean ± SD (n = 6). *, **, and *** show *p* < 0.05, *p* < 0.01, and *p* < 0.001, respectively. ns represents no significant difference.

The effect of HICC on intestinal inflammatory response was further studied in mice by assessing the expressions of inflammatory cytokines and chemokines from the mRNA levels or protein levels. Compared to the healthy control group, DSS-induced UC mice showed a severe inflammatory response in colonic tissue, which was evidenced by the significant elevation of gene expressions of pro-inflammatory cytokines (*IL-1β*, *TNF-α*, *IL-6*, and *IL-17a*) and chemokines (*MCP-1*, *Cxcl1*, *Cxcl2*, *Cxcl5*, and *Ccl7*) (Figure 2A,B). Except for *IL-1β*, *IL-17a*, and *Cxcl2*, these adverse alterations were significantly reversed in the HICC-treated mice (Figure 2A,B). Similarly, the protein levels of TNF-α, IL-1β, and IL-6 were significantly up-regulated in the DSS group, whereas they were all significantly prevented in both HICC and SASP-treated mice (Figure 2C–E). Lipopolysaccharides (LPS), one kind of endotoxin, are components of the outer wall of Gram-negative bacteria and typical pro-inflammatory substances. The results of serum LPS showed that DSS induction led to a significant increase in serum LPS, whereas these adverse changes were improved in both the HICC and SASP treatment groups (Figure 2F). These results suggested that HICC supplementation prevented the increase in serum LPS, which may further ameliorate the inflammatory response exacerbated by LPS. In summary, these data suggested that HICC could alleviate the inflammatory response in DSS-induced UC mice. 

### 3.3. HICC Attenuated the Oxidative Damage in DSS-Induced UC Mice

**Figure 3:**
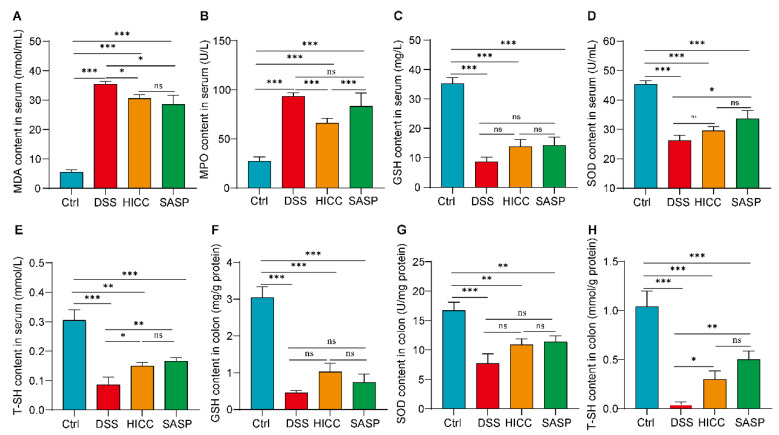
HICC attenuated oxidative damage in mice. (**A**) The serum MDA content. (**B**) The serum MPO content. (**C**) The serum GSH content. (**D**) The serum SOD content. (**E**) The serum T-SH content. (**F**) The colonic GSH levels. (**G**) The colonic SOD levels. (**H**) The colonic T-SH levels. Data were expressed as mean ± SD (n = 6). *, **, and *** show *p* < 0.05, *p* < 0.01, and *p* < 0.001, respectively. ns represents no significant difference.

Oxidative damage can exacerbate the progression of UC. When comparing the DSS-induced UC mice to the healthy control group, the contents of serum MDA and MPO were remarkably elevated, whereas the contents of GSH, SOD, and T-SH in serum were significantly decreased (Figure 3). After treatment with HICC, the DSS-induced elevation of serum MDA and MPO and the reduction in T-SH were significantly ameliorated (Figure 3). Thus, dietary intervention with HICC can partially alleviate DSS-induced oxidative damage, mainly by blocking MDA/MPO production and T-SH reduction. 

### 3.4. HICC Mitigated Intestinal Barrier Injury in DSS-Induced UC Mice

**Figure 4:**
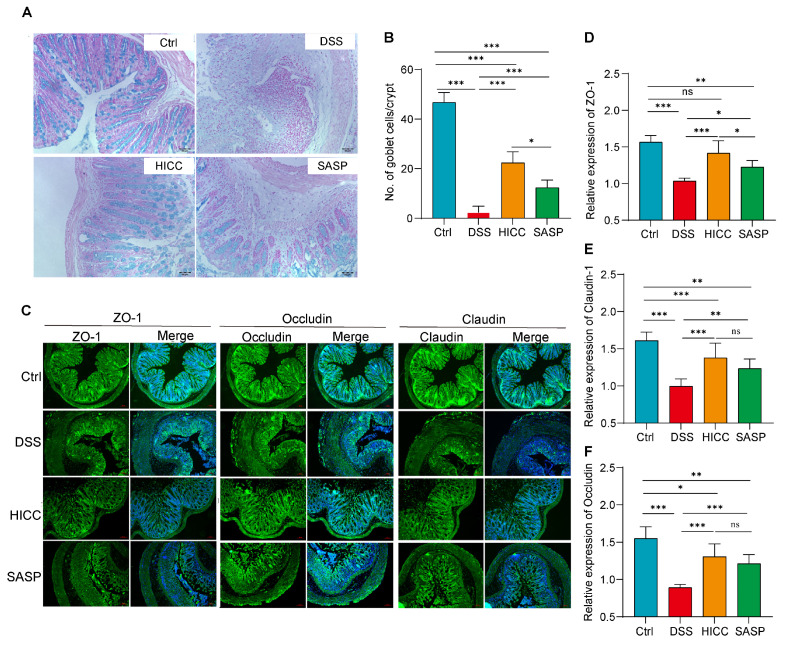
HICC mitigated intestinal barrier damage in mice. (**A**) The representative colonic images of Alcian blue staining. (**B**) The quantitation of goblet cells/crypt. (**C**) The representative immunofluorescence images of TJs (ZO-1, Occludin, and Claudin-1). (**D**) The quantitation of ZO-1 immunofluorescence. (**E**) The quantitation of Claudin-1 immunofluorescence. (**F**) The quantitation of Occludin immunofluorescence. Data were expressed as mean ± SD (n = 6). *, **, and *** show *p* < 0.05, *p* < 0.01, and *p* < 0.001, respectively. ns represents no significant difference.

The results of pathological analysis of the colon showed that the goblet cells almost completely disappeared and showed evident intestinal mucosal tissue damage in the colonic tissues of the DSS-induced mice (Figure 4A,B). After the dietary intervention of HICC and SASP, the intestinal tissue damage was somewhat alleviated, and the number of goblet cells was significantly increased (Figure 4A,B). The immunofluorescence results of mouse colonic tissues showed that the TJs (ZO-1, occludin, and claudin-1) were remarkably decreased in the mice induced by DSS application (Figure 4C–F). Their expressions were significantly restored after the HICC and SASP interventions (Figure 4C–F). In addition, as shown in Figure 4C–F, the colonic ZO-1 level in HICC-treated mice was significantly higher than that in the SASP-treated mice. The above data suggested that HICC could mitigate intestinal barrier damage.

### 3.5. HICC Modulated Intestinal Microbiota Structure in DSS-Induced UC Mice

**Figure 5:**
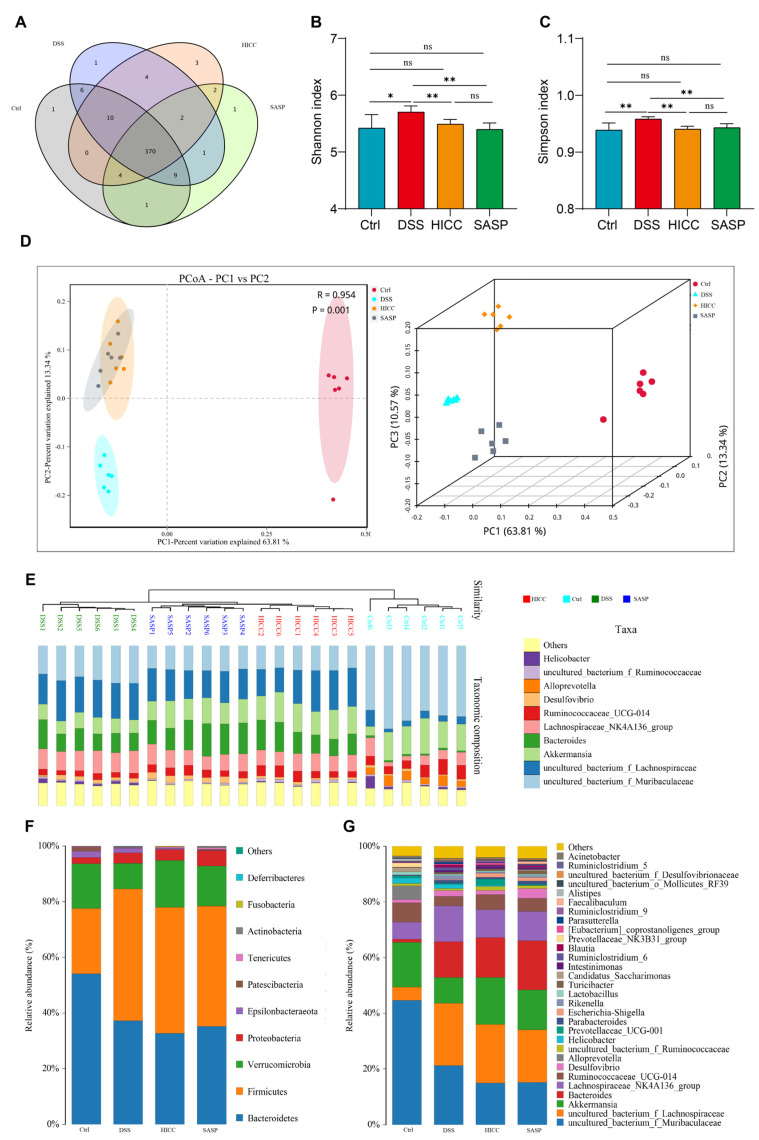
HICC modulated the intestinal microbiota in mice. (**A**) The Venn diagram of gut microbiota. The alpha-diversity of gut microbiota, including (**B**) Shannon index and (**C**) Simpson index. The beta-diversity of gut microbiota, including (**D**) PCoA plot and (**E**) UPGMA clustering tree. (**F**) The hierarchical clustering heatmaps of gut microbiota at (**F**) phylum level and (**G**) genus level. Data were expressed as mean ± SD (n = 6). * and ** show *p* < 0.05 and *p* < 0.01, respectively. ns represents no significant difference.

The result of the Venn diagram suggested that the number of OTUs in the Ctrl, DSS, HICC, and SASP groups were 401, 403, 395, and 390, respectively (Figure 5A). The Shannon and Simpson indexes of UC mice were significantly higher than that in the Ctrl group, suggesting that the diversity of gut microbiota was increased. However, it was significantly reduced after treatment with HICC and SASP (Figure 5B,C). The PCoA analysis showed that there was an apparent clustering in the Ctrl, DSS, HICC, and SASP groups. Moreover, the clustering of the DSS, HICC, and SASP groups was significantly far from the Ctrl group, suggesting different compositions of gut microbiota among the Ctrl and the other three groups (Figure 5D). In addition, the results of the UPGMA clustering tree showed that the four groups of the intestinal microbial communities were significantly different (Figure 5E).

At the top 10 phylum level (Figure 5F), the relative abundances of Firmicutes, Proteobacteria, and Epsilonbacteraeota were significantly increased in DSS-induced UC mice, whereas the abundance of Verrucomicrobia, Patescibacteria, and Bacteroidetes was significantly decreased, whereas the dietary application with HICC and SASP significantly prevented the increase in Epsilonbacteraeota and the decrease in Verrucomicrobia. At the top 30 family levels (Table S2, Figure S2A), DSS induction caused an increase in 9 families and a decrease in 9 families. After application with HICC, the relative abundance of seven families was greatly reversed as compared to the UC model mice, including five increased families induced by DSS (*Bifidobacteriaceae*, *Burkholderiaceae*, *Rikenellaceae*, *Clostridiales_vadinBB60_group*, and *Tannellaceae*) and two decreased families induced by DSS (*Clostriiaceae1* and *Prevotelaceae*). In addition, five families (including *Aeromonas*, *Enterobacteriaceae*, *Fusobacteriaceae*, *Ruminococcaceae*, and *Shewanellaceae*) were significantly increased in HICC-treated mice. At the top 30 genus levels (Figure 5G, Table S3, and Figure S2B), compared to the control mice, the abundance of 21 genera were greatly changed in DSS-induced UC mice, including 13 increased genera and 8 decreased genera. After supplementation with HICC, five DSS-increased genera (*Blautia*, *Parabacteroides*, *Parasutterella*, *Rikenella*, and *Ruminiclostridium_6*) and one DSS-decreased genus (*Alistipes*) were significantly reversed. In addition, the abundance of *uncultured_bacterium_f_Muribaculaceae* was further decreased, and the *Intestinimonas* abundance was further increased in the HICC group. In addition, six newly increased genera were observed in the HICC treatment group (e.g., *Prevotellaceae_UCG-001*, *Ruminiclostridium_5*, and *Ruminiclostridium_9*). 

**Figure 6:**
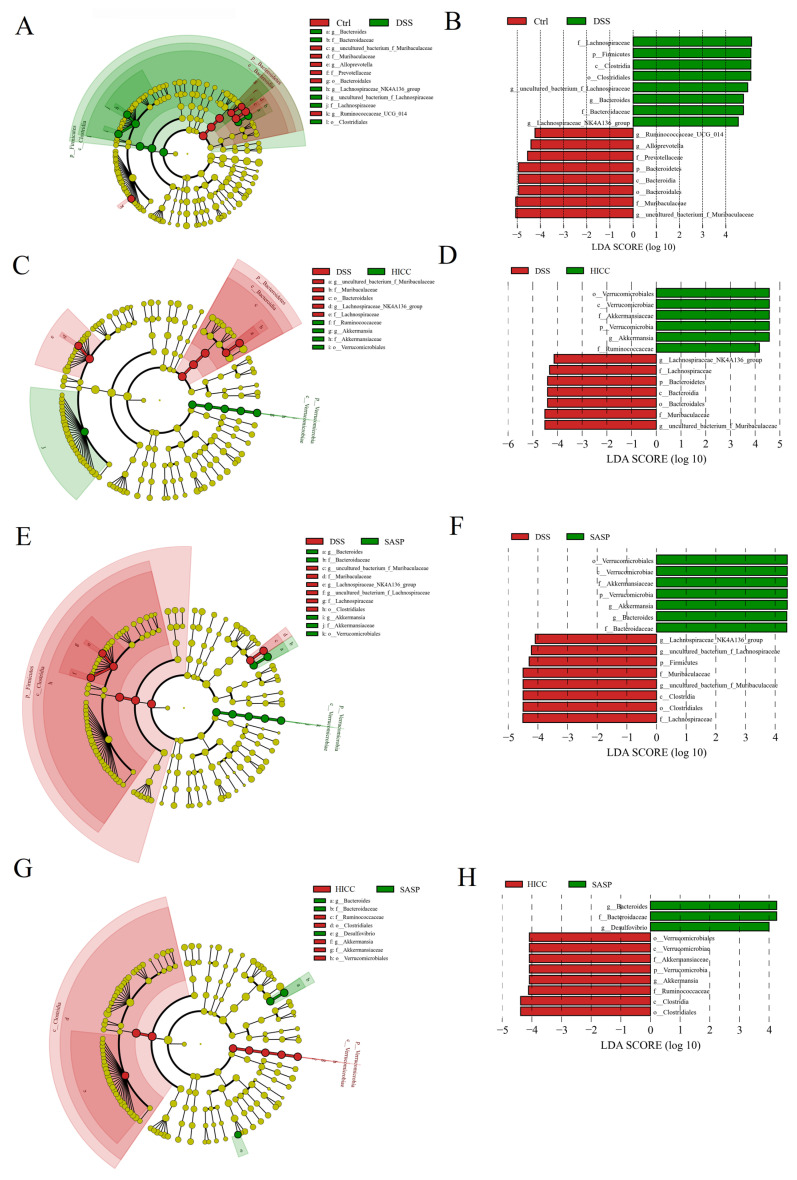
The LEfSe results of gut microbiota from the phylum and the genus levels. (**A**,**B**) Ctrl vs. DSS; (**C**,**D**) Ctrl vs. HICC; (**E**,**F**) DSS vs. SASP; (**G**,**H**) HICC vs. SASP. Only the LDA scores above 4.0 are shown.

The LefSe analysis was performed to further study the specific bacterial taxa (from phylum to genus level) among different groups (Figure 6). When comparing Ctrl and DSS groups, the *Bacteroidetes Bacteroidia*, *Bacteroidales*, *Prevotellaceae*, *Muribaculaceae*, *Ruminococcaceae_UCG_014*, and the *Alloprevotella uncultured_bacterium_f_Muribaculaceae* were the specific bacterial taxa in the Ctrl group; the Firmicutes *uncultured_ bacterium_f_ Lachnospiraceae*, *Clostridia*, *Clostridiales*, *Lachnospiraceae*, *Bacteroidaceae*, and *Lachnospiraceae_NK4A136_group* were the specific bacterial taxa in the DSS group. Compared with the DSS groups, the specific bacterial taxa in the HICC group were *Verrucomicrobia*, *Verrucomicrobiae*, *Verrucomicrobiales*, *Akkermansiaceae*, *Ruminococcaceae*, and *Akkermansia*. Between the HICC and SASP groups, the specific bacterial taxa in the SASP group were *Bacteroidaceae*, *Bacteroides*, and *Desulfovibrio*; the specific bacterial taxa in the SASP group were *Verrucomicrobia*, *Verrucomicrobiae*, *Clostridia*, *Verrucomicrobiales*, *Clostridiales*, *Akkermansiaceae Ruminococcaceae*, and *Akkermansia*. Taken together, the major biomarkers of the gut microbiota of DSS-induced colitis mice were *Lachnospiraceae* and *Bacteroidales*, whereas the major biomarkers in mice treated with HICC were *Akkermansiaceae* and *Ruminococcaceae*.

### 3.6. The Relationship between the Gut Microbiota and UC-Related Pathological Parameters

**Figure 7:**
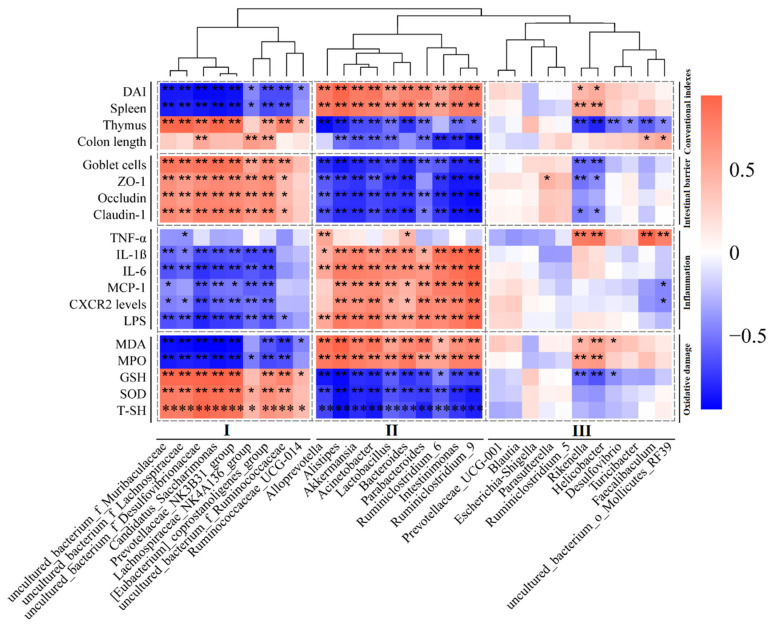
The relationship between the gut microbiota and UC-related pathological parameters based on the analysis of Pearson correlation coefficients. * and ** show *p* < 0.05 and 0.01, respectively.

The relationship between the gut microbiota and UC-related pathological parameters was studied using RStudio software by calculating Pearson’s correlation coefficient (Figure 7). In this study, the UC-related pathological parameters belonged to conventional indexes (DAI, spleen and thymus indexes, and colon length), intestinal barrier (goblet cells, occludin, ZO-1, and claudin-1), inflammation factors (proinflammatory cytokines, chemokines and LPS), and oxidative damage (oxidative mediators and antioxidant mediators). The heatmap results suggested that the genus levels of the top 30 bacteria can be clustered into three groups, including group I (9 genera), group II (10 genera), and group III (11 genera). Most group I bacteria were partly positively associated with the pathological index that could ameliorate UC (e.g., enhancing gut barrier function, ameliorating inflammation, and alleviating oxidative damage) and partly negatively associated with the pathological index aggravating UC (e.g., increased intestinal permeability, inflammation, and oxidative damage). On the contrary, most of the group II bacteria were partly negatively associated with the pathological parameters that could ameliorate UC and partly positively associated with the pathological index that aggravating UC. Most of the bacteria in group III showed almost no significant correlation with UC-related pathological parameters when compared to the bacteria in group II and group III.

## 4. Discussion

There is substantial evidence that probiotics can relieve colitis [22]. However, the essential condition for probiotics to exert their biological function is to remain active after reaching the host gastrointestinal tract [16]. Therefore, it poses great challenges for the processing, transportation, and storage of probiotic products. Compared to probiotics, inactivated probiotics (potential postbiotics) have more flexibility and competitive advantage due to their safety, storage stability, and better accessibility [26]. Research has shown that some inactivated microorganisms even have better beneficial functions than live bacteria. For example, compared to live *Akkermansia*, dietary intervention with inactivated *Akkermansia* significantly improved the health of insulin-resistant patients [27]. The inactivated microorganisms belong to the category of postbiotics defined by the International Scientific Association of Probiotics and Prebiotics (ISAPP) [18]. Although the studies on postbiotics started relatively late, there is much literature on their prebiotic potential, such as the mitigative effect on diarrhea [28] and irritable bowel syndrome [29].

Nowadays, UC has become a global public health problem, especially in some newly industrialized countries. Inactivated microorganisms, also known as postbiotics, have become a microecological therapy to alleviate UC mainly through maintaining the balance of intestinal microbiota, enhancing gut barrier function, modulating local and systemic immunity, and inhibiting oxidative damage [30,31]. A previous study has indicated that CC is a potential probiotic to relieve UC [22]. However, whether the mitigative effect of CC on UC is partly attributed to its inactivated cells that contain physiologically active substances is unclear. Therefore, in this study, the protective effect of HICC on UC in mice was investigated. 

Inflammatory response is a common feature of patients with UC, suggesting that inhibiting inflammation is an available alternative means to alleviate UC. In this study, the anti-inflammatory effect of HICC was confirmed both in vitro and in vivo. The results showed that the cell fragments of HICC suppressed *IL-6* and *IL-1β* mRNA levels in inflammatory RAW 264.7 cells. Additionally, after application with HICC, the inflammation-related pathological parameters were alleviated in the UC mice, as evidenced by the mitigation of the shortened colon length, the decrease in DAI, the improvement of immune organ indexes, and the lower expressions of pro-inflammatory cytokines and chemokines, etc. Numerous studies have found that heat-inactivated bacteria are rich in a variety of physiologically active substances that can ameliorate inflammatory responses, such as peptidoglycan, lipoteichoic acid, and exopolysaccharide. For example, the peptidoglycan derived from *Lactobacillus plantarum* ATCC 14917 inhibited IL-12 production in bacteria-induced macrophages [32]. The lipoteichoic acid of *Lactobacillus plantarum* alleviated Poly I:C-induced IL-8 production in porcine intestinal epithelial cells [33]. The exopolysaccharide derived from *Lactobacillus acidophilus* 20079 inhibited the NF-κB inflammatory pathways in human colorectal cancer [34]. Therefore, it can be speculated that the potential physiologically active substances in heat-inactivated microorganisms might be diverse, and the specific substance exerting anti-inflammatory effects in HICC needs to be further investigated.

The long-term chronic inflammatory response may lead to oxidative damage, which is also one of the common symptoms of colitis patients. Previous studies showed that patients with IBD show signs of increased oxidative damage, which may be partly attributed to the release of oxidant molecules released during chronic inflammatory response [35]. Conversely, oxidative damage may be due to the excessive production of reactive oxygen species, which may exacerbate inflammation and further tissue damage [36]. Therefore, the relationship between inflammation and oxidative damage is bidirectional, i.e., the inflammatory response may lead to oxidative damage, but it may also be the result of oxidative stress. Thus, alleviating oxidative damage may be an effective way to alleviate the inflammatory response, which may further alleviate UC. In the present study, the oxidative-damage-related indexes in the DSS group (e.g., the increased levels of serum MDA and MPO) were significantly attenuated after the supplementation with HICC. In addition, the depletion of reactive oxygen species to alleviate oxidative damage can also ameliorate UC by regulating the intestinal microbiota. This is because the excessive accumulation of reactive oxygen species can promote the growth of pro-inflammatory microorganisms that prefer a microaerobic environment [37]. These data suggested that the UC mitigating effect of HICC may be partly attributed to its ability to alleviate oxidative damage.

The composition disorder of gut microbiota may also promote the development of UC because it could trigger inflammatory responses and cause gut barrier damage [38]. For example, harmful gut microbiota (e.g., LPS and hydrogen sulfide-producing bacteria) are associated with gut inflammation and may aggravate the development and progression of IBD [39]. The present study showed that some pro-inflammatory bacteria (e.g., *Desulfovibrio*) were significantly increased in the DSS-induced UC mice, whereas they were suppressed after treatment with HICC. On the contrary, the beneficial gut microbiota (e.g., *Lactobacillus* and *Akkermansia*) can exert various healthy functions on the host. For example, both *Akkermansia* [40] and *Lactobacillus* [41] are potential anti-inflammatory bacteria because they are beneficial for the production of anti-inflammatory metabolites (e.g., short-chain fatty acids). In addition, a prior study has reported that *Akkermansia* can enhance the gut barrier function by enhancing the mucins secretion ability of goblet cells [42]. The results of this study indicated that the abundance of *Akkermansia* and *Lactobacillus* was higher in the HICC-treated mice than in the UC model mice. Meanwhile, the intestinal inflammatory response (the increase in proinflammatory cytokines and chemokines) was partly suppressed, and the intestinal barrier function (the levels of occludin, ZO-1, and claudin) was enhanced in the HICC-treated mice as compared with the DSS-indued mice. A healthy intestinal barrier function is essential, as it can selectively allow the passage of nutrients and water while blocking the entry of harmful substances (such as proinflammatory stimuli, such as bacteria and LPS) into the body. When the gut barrier is damaged, patients are often accompanied with IBD [43]. Therefore, treatment with HICC to strengthen the intestinal barrier function was also important to ameliorate UC.

Taken together, the supplementation of HICC could ameliorate UC in mice via multiple ways, mainly including ameliorating the colon inflammatory response, attenuating the oxidative and gut barrier damages, and modulating the gut microbiota. In fact, UC is the result of a combination of pathogenic factors. Thus, there may be an interaction relationship among the above-mentioned ways. 

## 5. Conclusions

Based on the analyses of the inflammatory response, oxidative damage, gut barrier, and gut microbiota, the present study demonstrated that HICC is a potential postbiotic in alleviating UC, which further suggests that HICC has the potential to be used as a dietary supplement for the relief of UC. However, due to the complex components of HICC, future studies must explore the specific physiological active substances that could relieve UC in HICC-treated mice.

## Data Availability

The data presented in this study are available on request from the corresponding author.

## Supplementary Materials

The following supporting information can be downloaded at: https://www.mdpi.com/article/10.3390/nu15122746/s1, Figure S1: The cell fragments of heat-inactivated *Companilactobacillus crustorum* MN047 (HICC) relieved inflammation in LPS-induced RAW 264.7 cells; Figure S2: The hierarchical clustering heatmap of the gut microbiota at (A) family and (B) genus levels; Table S1: The primer sequences of targeted genes used for quantitative real-time PCR; Table S2 Effects of heat-killed *Companilactobacillus crustorum* MN047 on the relative abundance of gut microbiota at family level (Top 30) in DSS-induced colitis mice; Table S3 Effects of heat-killed *Companilactobacillus crustorum* MN047 on the relative abundance of gut microbiota at genus level (Top 30) in DSS-induced colitis mice.
