# Supplementary material for: The Protective Effect of Heat-Inactivated Companilactobacillus crustorum on Dextran Sulfate Sodium-Induced Ulcerative Colitis in Mice
Source: Nutrients. 2023 Jun 14;15(12):2746. doi: 10.3390/nu15122746 (PMC10305042; doi:10.3390/nu15122746)
Supplement: Supplementary file 1 [file nutrients-15-02746-s001.zip › nutrients-2445715-supplementary.pdf]

The protective effect of heat-inactivated *Companilactobacillus crustorum* on dextran sulfate sodium-induced ulcerative colitis in mice

Materials and Methods

1.1 The preparation of HICC cell fragments

After the preparation of HICC, the HICC was freeze-grinded (-10 °C, the homogeneous speed of 20 m/s, 3 min) and freeze-dried to prepare cell fragments.

1.2 Cell culture and treatment

RAW 264.7 cells were cultured in DMEM medium containing 10% fetal bovine serum and 1% double antibodies (100 U/mL penicillin, 100 μg/mL streptomycin) at 37 °C and 5% CO_2. Then the cells with logarithmic growth phase were inoculated into a 6-well plate and cultured to 80% fusion. Next, the cells were divided into Cltrl group, LPS group (the cells were treated with 1 μg/mL of LPS for 24 h) and cell fragment of HICC (the cells were treated 1 μg/mL of LPS and 200 mg/L of cell fragments of HICC for 24 h). After treatment, the cells were collected after centrifugation (3000*g*, 5 min). Then the total RNA was extracted from the collected cells and reverse transcribed into cDNA for RT-qPCR analysis. The primer sequences of the targeted genes were expressed in Table S1.

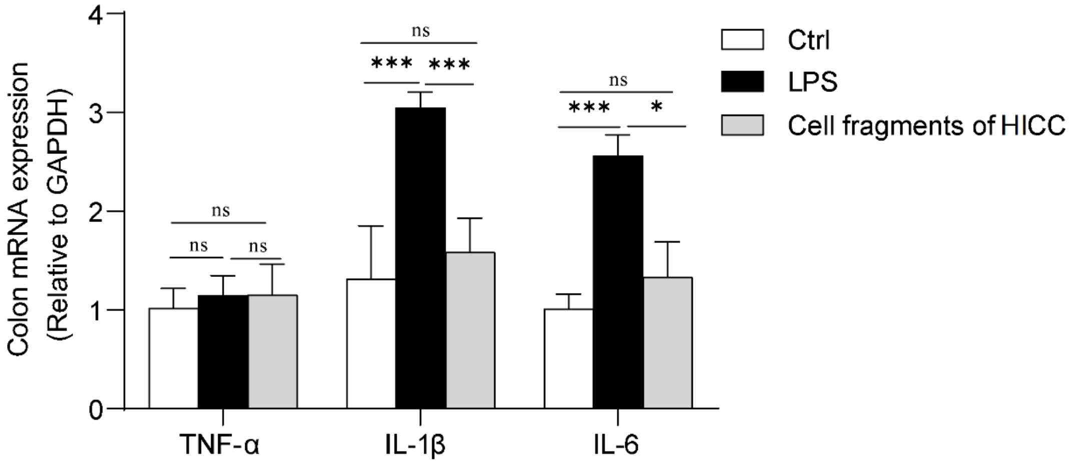

Figure S1. The cell fragments of heat-inactivated *Companilactobacillus crustorum* MN047 (HICC) relieved inflammation in LPS-induced RAW 264.7 cells. The mRNA levels of *TNF-α*, *IL-6*, and *IL-1β*. Data were expressed as mean ± SD (n = 6). * and *** indicate $P < 0.05$ and $P < 0.001$, respectively. ns represents no significant difference.

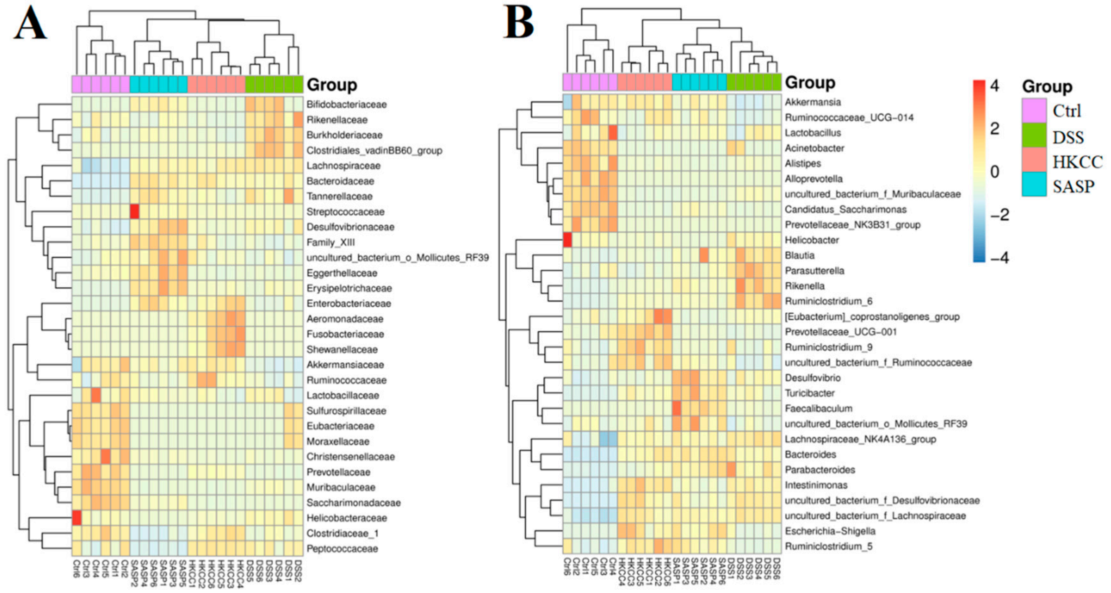

Figure S2. The hierarchical clustering heatmap of the gut microbiota at (A) family and (B) genus levels.

Table S1 The primer sequences of targeted genes used for quantitative real-time PCR

Target gene	Forward primer (5'→3')	Revere primer (5'→3')
TNF-α	CACAGAAAGCATGATCCGCG	CCATTTGGGAACTTCTCATCCC
IL-1β	GATGAAGGGCTGCTTCCAAAC	CTGCGAGATTTGAAGCTGGATG
IL-6	AGTTCCTCTCTGCAAGAGACTTC	AGTCTCCTCTCCGGACTTGT
IL-17a	TGGACTCTCCACCGCAATGAAG	GCTTTCCCTCCGCATTGACAC
MCP-1	CAGGTCCCTGTCATGCTTCT	CCCATTCCTTCTTGGGGTCA
Cxcl1	TGCACCCAAACCGAAGTCAT	ACTTGGGGACACCTTTTAGCAT
Cxcl2	GAGGGTGAGTTGGGAACTAGC	TTCCATGAAAGCCATCCGACT
Cxcl3	TGAGGCAGTATTCCTTGGCTG	ACCGGCATGACCTTGTTTGT
Cxcl5	TCCTCAGTCATAGCCGCAAC	TAGCTTTCTTTTTGTCACTGCCC
Ccl7	CCCTGGGAAGCTGTTATCTTCA	CTCGACCCACTTCTGATGGG
GAPDH	AGGTCGGTGTGAACGGATTTG	TGTAGACCATGTAGTTGAGGTCA

Table S2 Effects of heat-killed *Companilactobacillus crustorum* MN047 on the relative abundance of gut microbiota at family level (Top 30) in DSS-induced colitis mice

Family levels	Groups			
	Ctrl	DSS	HICC	SASP
Aeromonadaceae	0.0000 ± 0.0000[a] [1)]	0.0000 ± 0.0000[a]	0.0017 ± 0.0008[b]	0.0000 ± 0.0000[a]
Akkermansiaceae	0.1600 ± 0.0548[b]	0.0922 ± 0.0098[a]	0.1690 ± 0.0170[a]	0.1440 ± 0.0195[a]
Bacteroidaceae	0.0115 ± 0.0014[a]	0.1282 ± 0.0295[b]	0.1429 ± 0.0351[bc]	0.1766 ± 0.0271[c]
Bifidobacteriaceae	0.0000 ± 0.0000[a]	0.0020 ± 0.0015[b]	0.0001 ± 0.0001[a]	0.0014 ± 0.0003[b]
Burkholderiaceae	0.0023 ± 0.0016[a]	0.0071 ± 0.0012[b]	0.0017 ± 0.0006[a]	0.0028 ± 0.0006[a]
Christensenellaceae	0.0011 ± 0.0005[b]	0.0002 ± 0.0001[a]	0.0001 ± 0.0000[a]	0.0002 ± 0.0001[a]
Clostridiaceae_1	0.0029 ± 0.0006[c]	0.0014 ± 0.0005[b]	0.0027 ± 0.0008[c]	0.0004 ± 0.0003[a]
Clostridiales_vadinBB60_group	0.0003 ± 0.0003[a]	0.0051 ± 0.0013[b]	0.0006 ± 0.0002[a]	0.0005 ± 0.0003[a]
Desulfovibrionaceae	0.0113 ± 0.0046[a]	0.0248 ± 0.0065[b]	0.0182 ± 0.0016[ab]	0.0372 ± 0.0090[c]
Eggerthellaceae	0.0012 ± 0.0004[a]	0.0006 ± 0.0001[a]	0.0005 ± 0.0002[a]	0.0041 ± 0.0010[b]
Enterobacteriaceae	0.0010 ± 0.0002[a]	0.0027 ± 0.0034[a]	0.0147 ± 0.0076[b]	0.0150 ± 0.0066[b]
Erysipelotrichaceae	0.0012 ± 0.0004[a]	0.0068 ± 0.0031[b]	0.0071 ± 0.0014[b]	0.0248 ± 0.0053[c]
Eubacteriaceae	0.0019 ± 0.0004[b]	0.0006 ± 0.0009[a]	0.0000 ± 0.0000[a]	0.0000 ± 0.0000[a]
Family_XIII	0.0021 ± 0.0003[a]	0.0016 ± 0.0004[a]	0.0022 ± 0.0009[a]	0.0049 ± 0.0004[b]
Fusobacteriaceae	0.0000 ± 0.0000[a]	0.0000 ± 0.0000[a]	0.0024 ± 0.0014[b]	0.0000 ± 0.0000[a]
Helicobacteraceae	0.0199 ± 0.0279[a]	0.0155 ± 0.0061[a]	0.0040 ± 0.0018[a]	0.0008 ± 0.0005[a]
Lachnospiraceae	0.1166 ± 0.0652[a]	0.3687 ± 0.0229[b]	0.3192 ± 0.0514[b]	0.3011 ± 0.0259[b]
Lactobacillaceae	0.0097 ± 0.0064[a]	0.0058 ± 0.0036[a]	0.0043 ± 0.0020[a]	0.0057 ± 0.0010[a]
Moraxellaceae	0.0069 ± 0.0012[b]	0.0024 ± 0.0037[a]	0.0000 ± 0.0000[a]	0.0000 ± 0.0000[a]
Muribaculaceae	0.4465 ± 0.0433[c]	0.2121 ± 0.0226[b]	0.1486 ± 0.0079[a]	0.1516 ± 0.0069[a]
Peptococcaceae	0.0019 ± 0.0010[b]	0.0022 ± 0.0005[b]	0.0028 ± 0.0001[b]	0.0007 ± 0.0001[a]

Family levels	Groups			
	Ctrl	DSS	HICC	SASP
Prevotellaceae	0.0716 ± 0.0186[c]	0.0032 ± 0.0015[a]	0.0235 ± 0.0025[b]	0.0051 ± 0.0009[a]
Rikenellaceae	0.0100 ± 0.0029[a]	0.0175 ± 0.0046[b]	0.0066 ± 0.0007[a]	0.0082 ± 0.0022[a]
Ruminococcaceae	0.0967 ± 0.0191[ab]	0.0803 ± 0.0082[a]	0.1125 ± 0.0192[b]	0.0854 ± 0.0073[a]
Saccharimonadaceae	0.0147 ± 0.0030[c]	0.0016 ± 0.0003[ab]	0.0013 ± 0.0003[a]	0.0043 ± 0.0016[b]
Shewanellaceae	0.0000 ± 0.0000[a]	0.0000 ± 0.0000[a]	0.0019 ± 0.0011[b]	0.0000 ± 0.0000[a]
Streptococcaceae	0.0000 ± 0.0000[a]	0.0000 ± 0.0000[a]	0.0002 ± 0.0001[a]	0.0072 ± 0.0103[a]
Sulfurospirillaceae	0.0029 ± 0.0006[b]	0.0010 ± 0.0015[a]	0.0000 ± 0.0000[a]	0.0000 ± 0.0000[a]
Tannerellaceae	0.0020 ± 0.0006[a]	0.0114 ± 0.0045[b]	0.0056 ± 0.0015[a]	0.0111 ± 0.0025[b]
uncultured_bacterium_o_Mollicutes_RF39	0.0021 ± 0.0008[a]	0.0016 ± 0.0006[a]	0.0025 ± 0.0009[a]	0.0046 ± 0.0014[b]

I) Data are expressed as mean ± standard deviation (n = 6). Significant differences ($P < 0.05$) are indicated between different lowercase letters in the same row. Significance analysis was performed using one-way analysis of variance (ANOVA), followed by Tukey’s test for multiple comparisons.

Table S3 Effects of heat-killed *Companilactobacillus crustorum* MN047 on the relative abundance of gut microbiota at genus level (Top 30) in DSS-induced colitis mice

Genus level	Group			
	Ctrl	DSS	HICC	SASP
Acinetobacter	0.0069 ± 0.0012[b I)]	0.0024 ± 0.0037[a]	0.0000 ± 0.0000[a]	0.0000 ± 0.0000[a]
Akkermansia	0.1600 ± 0.0548[b]	0.0922 ± 0.0098[a]	0.1690 ± 0.0170[b]	0.1440 ± 0.0195[b]
Alistipes	0.0077 ± 0.0024[b]	0.0014 ± 0.0004[a]	0.0012 ± 0.0003[a]	0.0006 ± 0.0002[a]
Alloprevotella	0.0493 ± 0.0131[b]	0.0002 ± 0.0003[a]	0.0000 ± 0.0000[a]	0.0000 ± 0.0000[a]
Bacteroides	0.0115 ± 0.0014[a]	0.1282 ± 0.0295[b]	0.1429 ± 0.0351[bc]	0.1766 ± 0.0271[c]
Blautia	0.0012 ± 0.0010[a]	0.0082 ± 0.0023[c]	0.0021 ± 0.0006[ab]	0.0054 ± 0.0044[bc]
Candidatus_Saccharimonas	0.0147 ± 0.0030[c]	0.0016 ± 0.0003[ab]	0.0013 ± 0.0003[a]	0.0043 ± 0.0016[b]
Desulfovibrio	0.0110 ± 0.0046[a]	0.0211 ± 0.0061[b]	0.0144 ± 0.0021[ab]	0.0348 ± 0.0088[c]
Escherichia-Shigella	0.0003 ± 0.0001[a]	0.0024 ± 0.0032[a]	0.0142 ± 0.0077[b]	0.0124 ± 0.0057[b]
Faecalibaculum	0.0003 ± 0.0001[a]	0.0007 ± 0.0004[a]	0.0007 ± 0.0002[a]	0.0093 ± 0.0034[b]
Helicobacter	0.0199 ± 0.0279[a]	0.0155 ± 0.0061[a]	0.0040 ± 0.0018[a]	0.0008 ± 0.0005[a]
Intestinimonas	0.0009 ± 0.0009[a]	0.0069 ± 0.0007[c]	0.0092 ± 0.0014[d]	0.0037 ± 0.0011[b]
Lachnospiraceae_NK4A136_group	0.0615 ± 0.0334[a]	0.1288 ± 0.0097[b]	0.1011 ± 0.0090[b]	0.1048 ± 0.0148[b]
Lactobacillus	0.0097 ± 0.0064[a]	0.0058 ± 0.0036[a]	0.0043 ± 0.0020[a]	0.0057 ± 0.0010[a]
Parabacteroides	0.0020 ± 0.0006[a]	0.0114 ± 0.0045[b]	0.0056 ± 0.0015[a]	0.0111 ± 0.0025[b]
Parasutterella	0.0023 ± 0.0016[a]	0.0071 ± 0.0012[b]	0.0017 ± 0.0006[a]	0.0028 ± 0.0006[a]
Prevotellaceae_NK3B31_group	0.0147 ± 0.0053[b]	0.0000 ± 0.0001[a]	0.0000 ± 0.0000[a]	0.0000 ± 0.0000[a]
Prevotellaceae_UCG-001	0.0076 ± 0.0055[a]	0.0030 ± 0.0016[a]	0.0234 ± 0.0025[b]	0.0051 ± 0.0009[a]
Rikenella	0.0020 ± 0.0008[a]	0.0136 ± 0.0042[ab]	0.0045 ± 0.0005[b]	0.0067 ± 0.0020[c]
*Ruminiclostridium*_5	0.0013 ± 0.0011[a]	0.0013 ± 0.0002[a]	0.0038 ± 0.0010[b]	0.0031 ± 0.0008[b]
*Ruminiclostridium*_6	0.0003 ± 0.0004[a]	0.0112 ± 0.0030[c]	0.0037 ± 0.0005[b]	0.0035 ± 0.0006[b]

Ruminiclostridium_9	0.0023 ± 0.0010[a]	0.0028 ± 0.0015[a]	0.0059 ± 0.0010[b]	0.0024 ± 0.0004[a]
Ruminococcaceae_UCG-014	0.0692 ± 0.0206[b]	0.0344 ± 0.0042[a]	0.0540 ± 0.0162[ab]	0.0481 ± 0.0089[ab]
Turicibacter	0.0004 ± 0.0002[a]	0.0045 ± 0.0021[b]	0.0058 ± 0.0014[b]	0.0126 ± 0.0031[c]
[Eubacterium]_coprostanoligenes_group	0.0024 ± 0.0012[a]	0.0031 ± 0.0003[a]	0.0067 ± 0.0034[b]	0.0017 ± 0.0005[a]
uncultured_bacterium_f_Desulfovibrionaceae	0.0003 ± 0.0001[a]	0.0037 ± 0.0005[c]	0.0037 ± 0.0012[c]	0.0023 ± 0.0009[b]
uncultured_bacterium_f_Lachnospiraceae	0.0476 ± 0.0280[a]	0.2244 ± 0.0207[b]	0.2109 ± 0.0436[b]	0.1884 ± 0.0135[b]
uncultured_bacterium_f_Muribaculaceae	0.4465 ± 0.0433[c]	0.2121 ± 0.0226[b]	0.1486 ± 0.0079[a]	0.1516 ± 0.0069[a]
uncultured_bacterium_f_Ruminococcaceae	0.0094 ± 0.0027[a]	0.0074 ± 0.0020[a]	0.0146 ± 0.0030[b]	0.0098 ± 0.0013[a]
*Uncultured_bacterium_o_Mollicutes_RF*39	0.0021 ± 0.0008[a]	0.0016 ± 0.0006[a]	0.0025 ± 0.0009[a]	0.0046 ± 0.0014[b]

I) Data are expressed as mean ± standard deviation (n = 6). Significant differences ($P < 0.05$) are indicated between different lowercase letters in the same row. Significance analysis was performed using one-way analysis of variance (ANOVA), followed by Tukey's test for multiple comparisons.
